# The impact of accreditation on continuous quality improvement process in undergraduate medical education programs: A scoping review

**DOI:** 10.12688/mep.20142.1

**Published:** 2024-03-20

**Authors:** Sateesh B Arja, Bobbie Ann White, Jabeen Fayyaz, Anne Thompson

**Affiliations:** 1 Medical Education Unit, Avalon University School of Medicine, Willemstad, Curacao, Netherlands Antilles; 2 Health Professions Education Program, MGH Institute of Health Professions Education, Boston, Massachusetts, USA; 3 SimKids, University of Toronto, Toronto, Canada

**Keywords:** Accreditation, quality improvement, CQI, quality assurance, educational processes, student outcomes

## Abstract

**Background:**

Accreditation in medical education has existed for more than 100 years, yet the impact of accreditation remains inconclusive. Some studies have shown the effects of accreditation on student outcomes and educational processes at medical schools. However, evidence showing the impact of accreditation on continuous quality improvement of undergraduate medical education programs is still in its infancy. This scoping review explores the impact of accreditation on continuous quality improvement (CQI).

**Methods:**

This scoping review followed the methodology of the Preferred Reporting Items of Systematic Reviews and the Meta-Analysis extension for scoping reviews (PRISMA-ScR) checklist outlined by Arksey and O'Malley (2005). Databases, including PubMed, Medline, ERIC, CINHAL, and Google Scholar, were searched to find articles from 2000 to 2022 related to the accreditation of undergraduate medical education programs and continuous quality improvement.

**Results:**

A total of 35 full-text articles were reviewed, and ten articles met our inclusion criteria. The quality assurance approach is based on meeting the standards of accreditation. The quality improvement approach is based on striving for excellence. Literature suggests a requirement to move from student outcomes to CQI measures. CQI requires everyone in the organization to take responsibility and accountability, considering quality as the result of every single step or process and leaders supporting improvements in data collection and data analysis for quality improvement.

**Conclusions:**

The literature on accreditation and CQI are limited in number. More research studies are required to enhance undergraduate medical education accreditation practices' value to medical students, educators, academic leaders, programs, and the public. It was recommended that medical schools embrace the culture and vision perpetuated by the CQI process.

## Introduction

Accreditation is expected to ensure the quality assurance of higher education ( Salto, 2018). According to the World Health Organization (WHO), regulatory bodies should play a central role in ensuring that public and private sector professionals are competent, sufficiently experienced, and adhere to agreed standards relative to the scope of practice and competency enshrined in regulation and legislative norms; countries should be supported in establishing or strengthening them to provide continuous updates to accreditation and credentialing ( WHO, 2016, p. 20).

The WHO emphasized the importance of accreditation and regulation. But only 49% of countries have access to accreditation bodies that can accredit medical schools ( Bedoll *et al.*, 2021). van Zanten *et al.* (2012, p.587) defined *accreditation* as a "review of an educational program conducted by a governmental organization or a private entity accountable at a government level based on publicized standards and predetermined protocols." Medical education accreditation has existed for over 100 years ( Akdemir *et al.*, 2018; Loddenkemper *et al.*, 2010). However, the evidence for the impact of accreditation and its outcomes is inconclusive ( Acevedo-De-Los-Ríos & Rondinel-Oviedo, 2022; Blouin *et al.*, 2018; Dattey *et al.*, 2014; Volkwein *et al.*, 2007). The literature regarding the impact of accreditation on medical schools' processes or quality improvement (QI) processes is still emerging, even though there are few articles on this topic.

The accreditation process is important as the citations/negative reports and site visits by the expert reviewers can bring curricular reforms and enhance the credibility of these programs ( White *et al.*, 2013). Accreditation can lead to a change process and impact medical schools' educational processes ( Arja *et al.*, 2021; Blouin *et al.*, 2018). The accreditation-related activities enable medical colleges to review and reassess their processes that improve medical education programs ( Blouin *et al.*, 2018).

It was identified that graduates from accredited medical schools are performing better in programs with Educational Commission for Foreign Medical Graduates (ECFMG) certification ( Tackett *et al.*, 2021; van Zanten, 2015). Thus, educational program quality appears to be directly related to student performance, suggesting that the accredited undergraduate medical education programs improved the quality of education offered to the students. Educational programs did benefit from engaging in the quality improvement processes ( Tackett *et al.*, 2021). The ECFMG announced that "for a medical school to achieve recognized accreditation status, the accrediting agency of the school must be reviewed and recognized by an external quality assurance organization. This quality assurance organization, in turn, must be reviewed and approved by ECFMG. Only medical schools accredited by an agency recognized by an organization approved by ECFMG will satisfy the requirements of the Recognized Accreditation Policy" ( ECFMG, 2022). The announcement by ECFMG has further increased the importance of the accreditation of medical education programs.

Also, there is an inherent belief that the accreditation of medical schools guarantees the quality of educational programs of medical schools. Despite the importance and emphasis on accreditation, there is no guarantee that it achieves the intended outcomes of accreditation and medical schools. Quality management aims to achieve accreditation goals through a variable blend of quality assurance (QA), QI, and quality control ( Akdemir *et al.*, 2018).

Continuous quality improvement (CQI), which originated in business as a method of quality control for services or products ( Radawski, 1999), is an ongoing cycle of organizational improvement that uses quality indicators ( Kritchevsky & Simmons, 1991). It is time to move from QA to QI across medical education programs ( Barzansky *et al.*, 2015). Barzansky *et al.* (2015) suggested having a periodic evaluation of programs in addition to the summative evaluations of the programs by accreditation bodies at fixed intervals. Barzansky *et al.* (2015) emphasized the importance of submitting annual reports by medical schools. The functional CQI process should be feasible to implement. It should focus on accreditation standards to improve the quality of educational programs and outcomes and avoid duplication of efforts ( Barzansky *et al.*, 2015).

In 2015, the Liaison Committee on Medical Education (LCME) began requiring internal CQI processes for medical programs to ensure compliance with accreditation standards ( Stratton, 2019). LCME added the language to its standards: strategic planning and CQI, which states that a medical school must engage in ongoing planning and CQI processes that establish short- and long-term goals for the educational program, resulting in the achievement of measurable outcomes that are used to improve the quality of the educational program. This should ensure effective oversight of the educational program’s compliance with accreditation standards ( LCME, 2022, p. 1).

This scoping review explores the impact of accreditation on the CQI process in undergraduate medical programs. The scoping review maps pertinent literature about a topic of interest ( Arksey & O’Malley, 2005). A scoping review is less likely to address a specific research question and even less likely to evaluate the quality of studies included in the scoping review ( Arksey & O’Malley, 2005). The evidence about accreditation and CQI in medical education programs is still in its infancy ( Akdemir *et al.*, 2020). This led us to choose a scoping review over a systematic review to explore the impact of accreditation on the CQI process in undergraduate medical education programs.

## Methods

### Research protocol

We followed the scoping review framework, the Preferred Reporting Items of Systematic Reviews, and the Meta-Analysis extension for scoping reviews (PRISMA-ScR) checklist ( Arksey & O’Malley, 2005; Tricco *et al.*, 2018). It was summarized by the Joanna Briggs Institute (2015) as a six-stage approach to conducting the scoping review as outlined:

1. Identify the research question,
2. Identify relevant studies/literature,
3. Select the literature,
4. Chart the data,
5. Collate, summarize, and report results; and
6. Consult (optional)

A scoping review has a broader scope than a systematic review ( Arksey & O’Malley, 2005). The scoping review aims to identify the breadth of the current literature regarding the research question and identify knowledge gaps ( Arksey & O’Malley, 2005).

### Information sources and search strategy

We searched PubMed, Medline, ERIC, CINHAL and Google Scholar (2000 to present, in-process & other non-Indexed citations) to identify articles addressing the impact of accreditation on CQI process in undergraduate medical education programs. Search strategies were developed by an academic health science librarian (LN) with input from the project leads (SA) and co-leads (AT and JF). The keywords used for the search were “quality improvement,” “accreditation,” “medical education,” and “medical students.” Please refer to Table 1. Searches were completed in December 2022 and limited to articles published between January 1, 2000, and October 31, 2022. The reason for selecting the year 2000 reflects the relevance of data in this topic. English-language limits applied to all databases, and any article retrieved in a language other than English was excluded.

**Table 1.** Research Consultation & Search Strategy with Librarian Lindsey Nichols.

Context	AND	Condition	AND	Population
“quality improvement” OR “continuous quality improvement” OR CQI OR “change management” OR "Quality Improvement"[Mesh] OR "Change Management"[Mesh]		accreditation OR "Accreditation"[Mesh]		undergraduate AND (“medical school” OR “medical education”) OR "Education, Medical"[MAJR] OR "Education, Medical, Undergraduate"[Mesh] NOT residen* NOT post-graduate*

Search Tips: Truncat* = any variation of root word “Phrase search” = search exact phrase **Research Question:** Impact of higher ed accreditation on undergraduate medical education and continuous quality improvement **Search Strategy Approach & Lore**      1.   Break question into topic chunks. See table below.            a.   PEO question = *examines relationship* between Exposure and Outcome.                   i.   You can start with PEO, end with PICO.            b.   CoCoPop question = *explores scope of literature* on Condition and Context.                   i.   You can start w CoCoPop, end with PICO.            c.   PICO(T) question = *compares intervention against intervention*, or compare intervention against lack of intervention.            d.   Many other question frameworks Googleable, particularly for qualitative questions. (SPICE! SPIDER! ECLIPSE!)      2.   Brainstorm synonyms.            a.   Search for MeSH (Medical Subject Headings describing content) in PubMed.      3. **Keyword Harvest**: Skim titles and abstracts. Add new keywords and MeSH / subject headings to table as you find them in search results.            a.   Keyword synonyms within same column = combine with OR (usually)            b.   Keywords across columns = combine with AND            c. **Reminder**: Different databases have different formatting for subject headings.                   i.   Most databases *do* have a subject heading thesaurus in the top banner.                   ii.   Use each database’s thesaurus to build a *slightly* different search strategy for each database.      4.   Continue modifying search and harvesting keywords and MeSH until results are highly relevant to topic.            a.   Apply limits to search: date of study publication, age of participant, language of article, etc. **Databases & Search Strategy** **Pubmed** Two starting searches below: one broad *excluding* outcomes, and one focused *including* outcomes. **A.   Focused Search *including* outcomes**            ((undergraduate[Title/Abstract] AND ("medical school"[Title/Abstract] OR "medical education"[Title/Abstract]) OR ("Education, Medical"[MAJR] OR "Education, Medical, Undergraduate"[Mesh])) AND            ("accreditation"[Title/Abstract] OR "Accreditation"[Mesh])) AND            (("quality improvement"[Title/Abstract] OR CQI[Title/Abstract] OR "change management"[Title/Abstract]) OR ("Quality Improvement"[Mesh] OR "Change Management"[Mesh])) **279 results, no limits** **B.   Broad Search *excluding* outcomes**            ((undergraduate[Title/Abstract] AND ("medical school"[Title/Abstract] OR "medical education"[Title/Abstract]) OR ("Education, Medical"[MAJR] OR "Education, Medical, Undergraduate"[Mesh])) AND            ("accreditation"[Title/Abstract] OR "Accreditation"[Mesh])) **5240 results, no limits** ERIC **Gray Literature Search: conference papers, reports, etc.** **Bonus Resources**      1. PubMed Three Search Approaches Handout.docx      2. Mastering Keyword Searching async workshop (self-enroll)            a.   Refresher on Boolean searching in Advanced Search of Pubmed, other subject databases.      3. PubMed Online Training from National Library of Medicine            a.   Quick tours in 1–3 mins; 60 min tutorials; 90–300 min online classes in how to search PubMed.      4. Zotero.org <- free online reference manager; 2-click citations and references in Word or Google Docs.            a.   Zotero’s own Help Documentation is easy to read, includes screenshots.            b.   Download 1) Zotero, and 2) Zotero Connector extension for your browser (Chrome, Safari, etc.).                   i.   Zotero Connector = one-click add online sources (including both metadata and PDFs or HTML full text) to your Zotero library.      5. Zotero D2L async workshop (self-enroll)            a.   Short videos, short quizzes, light reading. Leads you through installation, setup, basic and advanced features.

### Eligibility criteria and selection of sources

All abstracts were independently screened (by lead and co-lead, SA and JF). The articles resulting from the first database (Medline, PubMed, and ERIC), second database (CINAHL), and Google Scholar searches were screened for relevance by the principal investigator (SA), who was, in turn, monitored by another co-lead (JF). Both investigators screened titles and abstracts in a data sheet using Excel. A common consensus was required between SA and JF. If there was a disagreement, it was resolved by another co-lead (AT). At first, only titles and abstracts were reviewed. Only articles not excluded through the abstract review underwent full-text review. Primary research articles written in English were included if they:

1. Discussed the accreditation process.
2. Emphasized the impact of accreditation on the CQI process.
3. Involved undergraduate medical programs.

Articles were excluded if they discussed the QI curricula in postgraduate training or the implementation of ACGME guidelines for postgraduate training. All three investigators (SA, AT, and JF) discussed any discrepancies related to inclusion and exclusion.

### Data charting of selected articles and process

The principal investigator (SA) and co-investigator (JF) read full-text articles, extracted data, and charted detailed information in an Excel standardized data extraction sheet. A data extraction framework was created to present the data broadly, including author, year of publication, place of study, aim/purpose, methods, participants, intervention, outcome, and key findings. Another co-lead (AT) reviewed the data extraction sheet. Please refer to Table 2. 

**Table 2.** Scoping review-summary of articles.

First Author	year	Country	Title of the article	Study population	Aim	Methodology	Outcome measures	Results/conclusions
Akdemir	2020	Canada Netherlands	Evaluation of continuous quality improvement in accreditation for medical education	The CQI experiences with three accreditation systems from USA, Canada and Netherlands		Naturalistic utility-focused evaluation. Naturalistic approach connected to real-life experiences.		The gathering of evidence about accreditation systems and CQI in medical education seems to be still in its infancy. Further research is needed.
Blouin	2019	Canada	Quality improvement in medical schools: vision meets culture.	16 Canadian medical schools’ Academic leaders, formal teachers and clinical teachers	study explores whether QI practices are recognised as such in organisations not culturally QI-oriented. Specifically, it examines faculty members’ perceptions about the degree of QI implementation in their medical education programmes.	Respondents rate each statement on a 5-point Likert-type scale. Parametric statistical analysis (means and SDs).	questionnaire of the Malcolm Baldrige National Quality Award framework	Perceived QI implementation levels were low across programmes and for each category of respondents. This was especially true for the domains of ‘Strategy’, ‘Measurement/ analysis/knowledge management’ and ‘Operations’. Medical schools’ existing QI processes are not recognised as QI activities. For QI strategies to succeed, a programme’s culture must embrace QI. In the execution of their QI visions, medical schools should spend resources on embedding quality in the organisation culture in addition to strengthening existing QI practices.
Stansfield	2019	USA	Integration of Continuous Quality Improvement Methods into Annual Program and Institutional Evaluation	Wayne State University Office of Graduate Medical Education (WSU GME)	To improve the institutional- and program-level evaluation processes, to operationalize a culture of continuous quality improvement (CQI), and to increase the quality and achievement of action items	WSU GME phased the 4 CQI elements into the evaluation process at the program and institutional levels, including the annual program evaluation (APE) and the annual institutional review.		Using a systematic goal-setting process (SMART) and a systematic multi-source assessment process (dashboards), WSU GME has begun to successfully leverage widely accepted CQI methods to capitalize on program and institutional opportunities and strengths and to foster a more effective culture of educational improvement.
Stratton	2019	USA	Legitimizing Continuous Quality Improvement (CQI): Navigating Rationality in Undergraduate Medical Education		practical strategies to shield CQI from being passively dismissed, excessively routinized.	Commentary/ perspective		Ongoing improvement, rather than attainment of a static benchmark. Establish a Clear Purpose and coordinate data collection and reporting with existing scheduled activities, such as departmental reviews, university accreditation, or strategic planning. Secure Leadership Buy-in. Envision a Process. “Humanize” the Process. Share Results, Accountability and Scrutinize Measures & Outcomes.
Blouin	2018	Canada	Accreditation of Medical Education Programs: Moving from Student Outcomes to Continuous Quality Improvement Measures		Establishing the connection between accreditation and CQI and thereby improvement in medical education programs leading to better graduates.	perspective		Markers of effectiveness of accreditation reported in the literature focus on curricular transformations and the quality of programs’ output, including the number of curricular changes implemented by programs, organizational quality outcomes, and student outcomes. Markers of its impact need to include CQI-related measures. It is time to move away from a focus on student outcomes as measures of the impact of accreditation and embrace additional markers, such as indicators of organizational CQI orientation.
Nekdemir	2018	Australia	Accreditation as a quality improvement tool: is it still relevant?		Examines Australian Medical Council (AMC) standards.			Quality management aims to achieve the objectives of accreditation as effectively as possible through a variable mix of quality assurance, quality improvement and quality control. In the quality assurance approach, minimum standards are set to assure the quality of education and graduates produced. The legislation under which the AMC accredits programs requires this approach and focuses on compliance. The quality improvement approach is based on striving for excellence.
Ward	2018	USA	Implementing a Course Review Process for a Continuous Quality Improvement Model for a Medical School Curriculum.	Meharry Medical College School of Medicine.				Plan- Do- Study- Act Cycle for Learning and Improvement for continuous review of course content, outcomes, and evaluations. This process identifies strengths, challenges, and opportunities for innovative steps for continuous quality improvements to the curriculum.
Alrebish	2017	Saudi Arabia	Accreditation of medical schools in Saudi Arabia: A qualitative study		The aim of the study was to examine the purposes and outcomes of accreditation, and stakeholders' experience of accreditation in Saudi Arabia.	Thematic analysis approach. Triangulation of data. Literature review, analysis of accreditation documents, examined the outcome of accreditation process (pre and post) through stakeholders' experience of accreditation (learner, teacher, and academic leader perspectives)	The three-phase study: "Passing the exam" versus long-term benefit, generic versus specialized accreditation standards, and internal quality assurance and self-evaluation.	Sustainable accreditation means that there is a need to meet both the immediate accreditation standards (""the exam"") as well as establishing a basis for continuing quality improvement. Medical school leaders should “perceive” and “promote” the accreditation process as a journey for quality improvement, rather than as an exam that must be passed. Faculty development should be encouraged with the aim of establishing a culture of quality improvement rather than a guide on “how to be accredited”. The self-study process, with multiple stakeholder input, is key to building ownership in self-evaluation and goal setting within medical schools."
Shroyer	2016	USA	Drivers of Dashboard Development (3-D) A Curricular Continuous Quality Improvement Approach	SBU school of Medicine	Continuous quality improvement (CQI) settings, summary dashboard reports have been used to evaluate and improve performance.	This report represents a summary of the SBU School of Medicine Undergraduate Medical Education office’s Drivers of Dashboard Development (3-D) approach for continuous quality improvement.	Substantial improvements over time have been documented in KPIs including timeliness of clerkship grades, mid-clerkship feedback, student mistreatment policy awareness, and student satisfaction.	Stakeholder feedback indicates that the dashboards have provided useful information guiding data-driven curricular changes, such as integrating clinician–scientists as lecturers in basic science courses to clarify the clinical relevance of specific topics. The 3-D approach may be considered by UME programs as a template for providing faculty and leadership with a CQI framework to establish shared goals, document compliance, report accomplishments, enrich communications, facilitate decisions, and improve performance.
Barzansky	2015	USA, Canada, Taiwan, Korea	Continuous quality improvement in an accreditation system for undergraduate medical education: Benefits and challenges	National association of medical schools and several medical school accrediting bodies.	To identify the factors that affect the ability to implement a continuous quality improvement (CQI) process for the interval review of accreditation standards.	Case examples from the United States, Canada, the Republic of Korea and Taiwan, were collected and analyzed to determine the strengths and challenges of the CQI processes.		Accreditation standards so as to result in the improvement of educational quality and outcomes, be feasible to implement, avoid duplication of effort and have both commitment and resource support from the sponsoring entity and the individual medical schools. CQI can enhance educational program quality and outcomes, if the process is designed to collect relevant information and the results are used for program improvement. Medical schools should engage in interim review of their compliance with the accreditation standards of their country and act on the results in order to support a culture of continuous quality improvement (CQI).

### Ethical approval

This study was exempted from the research and ethics committee approval at Avalon University School of Medicine as this was a scoping review.

## Results

The first database search, on PubMed, Medline, and ERIC resulted in 279 abstracts. Of these 279 abstracts, 31 required a review of full-text articles, and one was a repeat. We excluded 247 articles based on graduate medical education or QI curriculum for residents, related to medical care, healthcare not in English, etc. Out of the 31 articles mandated for full-text review, 17 articles were categorized under general articles on accreditation, based on standards of accreditation and recent trends of accreditation, three articles were based on the impact of accreditation on educational processes, one article was based on the impact of accreditation on student outcomes, and ten articles described accreditation and CQI. The second database CINHAL resulted in nine abstracts. Out of the nine abstracts, three were removed as they were repeated from PubMed, and one was removed as it was published in a language other than English. The other five were also excluded as they were related to graduate medical education, education in general, QI in general education, or healthcare. The third database, Google Scholar search, resulted in 19,000 abstracts. But we reviewed only the initial 75 abstracts as Google Scholar was not returning many useful articles for this scoping review or the research question. Out of 75, three were repeats from PubMed. The other 68 articles were excluded as they were unrelated to this scoping review or the research question. Only four articles from Google Scholar required full-text review. They were related to accreditation and accreditation standards and had nothing to do with CQI. Only ten articles that described accreditation and CQI (PubMed) were included in the final review to answer the research question.

**Figure 1.**
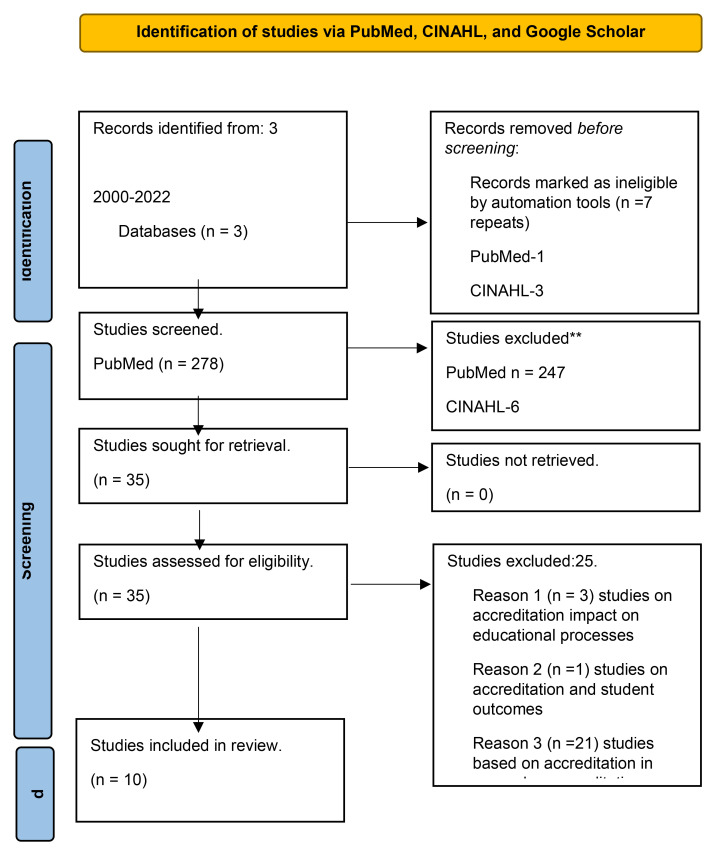
PRISMA flow diagram for “The impact of accreditation on continuous quality improvement process in undergraduate medical education programs: A scoping review”. *Adopted from:* Page MJ, McKenzie JE, Bossuyt PM, Boutron I, Hoffmann TC, Mulrow CD, *et al.* The PRISMA 2020 statement: an updated guideline for reporting systematic reviews. BMJ 2021;372: n71. doi: http://dx.doi.org/10.1136/bmj.n71. For more information, visit: http://www.prisma-statement.org/

In summary, we reviewed 35 full-text articles. Out of 35 articles, 21 concerned accreditation and its standards, including recent trends in medical education accreditation; three articles were related to accreditation and its impact on medical school education processes, one was related to accreditation and its impact on student outcomes, and ten published articles concerning accreditation and CQI. We were able to review another four articles found while retrieving these 35 articles. Of these four articles, two concern accreditation and its standards in general, one related to accreditation and its impact on medical school education processes, and two related to accreditation and its impact on student outcomes. Please refer to Figure 1.

The ten articles included for full review concerning the accreditation and CQI are as follows. Akdemir *et al.* (2018) defined QA and QI approaches in the accreditation of undergraduate medical education programs. After examining CQI's role in three accreditation systems, including the American and Canadian ones, the authors observed that CQI in undergraduate medical accreditation is still in its infancy ( Akdemir *et al.*, 2020). Sustainable accreditation means meeting the immediate accreditation standards and establishing a ground for CQI ( Alrebish *et al.*, 2017). It was advocated for moving from student outcomes to CQI measures ( Barzansky *et al.*, 2015; Blouin & Tekian, 2018). Ongoing improvement should be emphasized rather than the attainment of a static benchmark incorporating activities such as departmental reviews, university accreditation, or strategic planning ( Stratton, 2019). Blouin and Tekian (2018) suggested that the CQI should be one of the markers for accreditation impact if accreditation is intended to promote CQI.

Integrating CQI methods into annual programs and institutional evaluation is critical ( Stansfield *et al.*, 2019). It is also time for medical schools to change their approach toward CQI. In the execution of their QI visions, medical schools should spend resources on embedding CQI in the organization's culture ( Blouin, 2019). The Plan- Do- Study- Act cycle was implemented in one of the studies for learning and improvement because continuous review of course content, outcomes, and evaluations is a process that identifies strengths, challenges, and opportunities for innovative steps for CQI to the curriculum ( Ward *et al.*, 2018). Establishing CQI 3D dashboards incorporating the CQI framework was innovative ( Shroyer *et al.*, 2016). 

## Discussion

We categorized the articles into four themes as follows:

1. Accreditation and its standards in general,
2. Accreditation and its impact on student outcomes,
3. Accreditation and its impact on medical school's educational processes,
4. Accreditation and CQI.

In addition to accreditation and CQI findings, we would like to describe the themes that illustrate the breadth of literature.

### Accreditation and its impact on student outcomes

The publications on accreditation and student outcomes were Roy *et al.* (2020), Al-Eyadhy and Alenezi (2021), and Tackett *et al.* (2021). There was a positive correlation between the accreditation cycle and students' performance on national examinations among Canadian medical schools ( Roy *et al.*, 2020). Al-Eyadhy and Alenezi (2021) found that accreditation cycles were associated with increased student satisfaction rates. The pre-accreditation phase activities and the self-study process are essential triggers for QI practices related to accreditation. Tackett *et al.* (2021). identified a significant association between the accreditation status of the medical school and ECFMG certification rates. This suggests that program quality, in terms of student performance, may benefit from engagement in QI processes.

### Accreditation and its impact on medical schools’ educational processes

The publications about accreditation and its impact on medical school's educational process are Blouin (2020), Blouin *et al.* (2018), Arja *et al.* (2018), and Nora (2022). Blouin *et al.* (2018) and Arja *et al.* (2021) each identified processes that are impacted by accreditation, including governance, data collection and analysis, creation and revision of policies and procedures, curricular reforms, continuous quality improvement, and faculty members' engagement in the educational program. Blouin (2020) identified new themes impacted by accreditation, including the undergraduate medical education (UME) program process, UME program quality, QA, QI, and research.

### Accreditation and CQI

The accreditation systems are based on either the QA or QI approaches. In the QA approach, minimum standards are met to ensure the quality of education and training and meet the accreditation standards ( Akdemir *et al.*, 2018). The QI method is based on striving for excellence of standards. The balanced accreditation must include both QA and QI. Medical school leaders should use the accreditation process as a journey for QI rather than as an evaluation that must be passed ( Alrebish *et al.*, 2017). There is considerable debate about moving the accreditation systems from student outcomes to the CQI measures ( Barzansky *et al.*, 2015; Blouin & Tekian, 2018). Accreditation should not be all about ticking the standards checklist at one point in time and should emphasize ongoing improvements and CQI. CQI can enhance program quality and outcomes if the process is designed to collect appropriate information and the results are used for program development. Medical schools should intermittently review their compliance with the accreditation standards, submit annual reports to the accreditation bodies, and act on the results to support CQI ( Barzansky *et al.*, 2015). The ongoing improvement should include departmental reviews or reviews based on short-term and long-term measurable outcomes of strategic planning ( Stratton, 2019). CQI should be like envisioning and humanizing the process, sharing results with all stakeholders, expecting accountability from all stakeholders, and monitoring measures and outcomes ( Stratton, 2019).

Perceived QI implementation levels were low across programs in the 'strategy,' 'measurement/analysis/knowledge management’, and 'operations' domains ( Blouin, 2019). Medical schools' existing QI practices should be recognized as QI activities. In the execution of their QI visions, medical schools should spend resources on embedding quality in the organization's culture and reinforcing existing QI practices. Shroyer *et al.* (2016) showed an example of CQI 3D dashboards incorporating the CQI framework, which includes establishing shared goals, document compliance, reporting accomplishments, enriching communications, facilitating decisions, and improving performance. For QI strategies to succeed, a program's culture must embrace QI ( Blouin, 2019). With multiple stakeholder inputs, the self-study and evaluation process are key to building ownership in self-evaluation and goal setting within medical schools. The leaders and programs should invest resources and time for CQI. Medical schools should meet the accreditation standards and there should be an ongoing improvement of the program against the measurable outcomes/strategic plan. The CQI should be integrated into annual institutional and program reviews ( Stansfield *et al.*, 2019).

## Conclusions

Minimal evidence about accreditation and CQI exists. The number of studies in this field are limited and require further investigation about the impact of accreditation on CQI. Accreditation standards should align with CQI. One way to do this is to build a culture and vision that includes CQI in Medical Education. Finally, educational programs and leaders should adopt resources and faculty development promoting CQI that addresses larger issues and accreditation simultaneously.

## Data Availability

The data for this article consists of bibliographic references, which are included in the References section. Figshare: “The PRISMA checklist-The impact of accreditation on continuous quality improvement process in undergraduate medical education programs: A scoping review.”, https://doi.org/10.6084/m9.figshare.25021646.v1 ( Arja, 2024). Data are available under the terms of the Creative Commons Zero "No rights reserved" data waiver (CC0 1.0 Public domain dedication).
