# Supplementary material for: The impact of accreditation on continuous quality improvement process in undergraduate medical education programs: A scoping review
Source: MedEdPublish (2016). 2024 May 23;14:13. Originally published 2024 Mar 20. [Version 2] doi: 10.12688/mep.20142.2 (PMC11126904; doi:10.12688/mep.20142.2)
Supplement: Peer review history

# Reviewer report: Reviewer response for version 2

**DOI:** 10.21956/mep.21891.r37162

**Published:** 2024-05-30

**Authors:** Mohamed Elhassan Abdalla

**Affiliations:** 1 Faculty of Education & Health Services, University of Limerick, Limerick, County Limerick, Ireland

The authors have addressed the comments. I am happy to approve the article.

Are the rationale for, and objectives of, the Systematic Review clearly stated?

No

Is the statistical analysis and its interpretation appropriate?

No

Are sufficient details of the methods and analysis provided to allow replication by others?

Partly

Are the conclusions drawn adequately supported by the results presented in the review?

Yes

Reviewer Expertise:

Medical Education

I confirm that I have read this submission and believe that I have an appropriate level of expertise to confirm that it is of an acceptable scientific standard.

# Reviewer report: Reviewer response for version 2

**DOI:** 10.21956/mep.21891.r37163

**Published:** 2024-05-24

**Authors:** Pathiyil Ravi Shankar

**Affiliations:** 1 IMU Center for Education, International Medical University, Kuala Lumpur, Malaysia

The authors have addressed my comments. I approve this article.

Are the rationale for, and objectives of, the Systematic Review clearly stated?

Yes

Is the statistical analysis and its interpretation appropriate?

I cannot comment. A qualified statistician is required.

Are sufficient details of the methods and analysis provided to allow replication by others?

Yes

Are the conclusions drawn adequately supported by the results presented in the review?

Yes

Reviewer Expertise:

Health professions education

I confirm that I have read this submission and believe that I have an appropriate level of expertise to confirm that it is of an acceptable scientific standard.

# Reviewer report: Reviewer response for version 1

**DOI:** 10.21956/mep.21567.r36440

**Published:** 2024-05-22

**Authors:** David Rojas

**Affiliations:** 1 University of Toronto, Toronto, Ontario, Canada

It's crucial for the author to incorporate references that are more closely tied to medical education, especially those pertaining to accreditation. While references from other fields can be informative, the primary aim of this article is to establish the current status of continuous quality improvement and accreditation in the context of medical education. Therefore, medical education references would be more appropriate and beneficial. 

 It would be beneficial for the author to revisit the articles from Van Zanten and Taccket 2021. These articles only show a moderate effect from those accredited schools, and also elaborate on all the cofounders and why the results are not conclusive. This should be clearly stated in the article. Moreover, the announcement of the ECFMG to only allow applications from those trainees who have graduated from accredited institutions (with the WFME stamp) changed over the last year. Now, that is no longer a requirement to apply for the ECFMG certification. Reflecting this in the text will add value to the article. 

 There is also evidence that the accreditation process Does not trigger curricular reform. Please review [1]

 The discussion section of the article seems to cover a wide range of topics, not just the ones identified via the scoping review. While I understand the authors' intentions, this approach may pose some limitations regarding the reporting standard for scoping reviews. For instance, how did the authors select the information they are presenting in the discussion? If the article is about accreditation and continuous quality improvement processes, why start with the impact on “student outcomes and educational processes”? Are these the only articles that cover these topics? Were they also identified via the scoping review search? A more focused and structured approach could help address these questions. I would refrain from covering areas outside the focus of the scoping review or aim to create a better argument and offer a better explanation of why this information is being presented in the article. 

 Your research has the potential to significantly contribute to our understanding of the accreditation and CQI process. By structuring the discussion section around the main focus of the article, you can further enhance its impact and inspire further research in this Area. 

 One key element that needs to be added to the current manuscript is the overall analysis of the literature found. The authors have found 10 articles, and they do a good job of describing them. However, when the articles are analyzed together - what suggestions do we have? What is the learning from the 10 articles (learning is different from describing the articles)? This approach aligns more with what is expected at the end of a scoping review. Having a section dedicated to the overall learning from the literature found will add robustness to the approach presented here.

Are the rationale for, and objectives of, the Systematic Review clearly stated?

Yes

Is the statistical analysis and its interpretation appropriate?

Partly

Are sufficient details of the methods and analysis provided to allow replication by others?

Yes

Are the conclusions drawn adequately supported by the results presented in the review?

Partly

Reviewer Expertise:

Program Evaluation, Medical Education Accreditation, Systems thinking, Techonolgy enhanced medical educaiton

I confirm that I have read this submission and believe that I have an appropriate level of expertise to confirm that it is of an acceptable scientific standard, however I have significant reservations, as outlined above.

## References

[1] : The influence of accreditation on educational change in U.S. medical schools. *Acad Med* .1997;72(12) : 10.1097/00001888-199712000-00029 1127-33 10.1097/00001888-199712000-00029 9435724

# Reviewer report: Reviewer response for version 1

**DOI:** 10.21956/mep.21567.r36439

**Published:** 2024-05-17

**Authors:** Mohamed Elhassan Abdalla

**Affiliations:** 1 Faculty of Education & Health Services, University of Limerick, Limerick, County Limerick, Ireland

General Comment: The topic is important and can contribute to the discussion on the expected role of accreditation. 

 Specific Comments:

 Abstract: The section needs more information to be informative, especially the results. 

 Introduction: The section provides a good overview of the accreditation process and quality improvement in medical education. The section failed to build a good rationale for the research, it needs to present what is there in the literature in relation to role of accreditation in quality improvement, what is is missing and how the current work can lead to cover that missings.

 Methods: Will benefit from more information in each of the steps. The eligibility criteria is broad. No description on the data analysis . 

 Results: This is a very short section. Authors can do well if they review the different guidelines on reporting the results for a scoping review. 

 Discussion: It may change if the results section has to change based on the comments above.

Are the rationale for, and objectives of, the Systematic Review clearly stated?

No

Is the statistical analysis and its interpretation appropriate?

No

Are sufficient details of the methods and analysis provided to allow replication by others?

Partly

Are the conclusions drawn adequately supported by the results presented in the review?

Yes

Reviewer Expertise:

Medical Education

I confirm that I have read this submission and believe that I have an appropriate level of expertise to confirm that it is of an acceptable scientific standard, however I have significant reservations, as outlined above.

# Reviewer report: Reviewer response for version 1

**DOI:** 10.21956/mep.21567.r36601

**Published:** 2024-04-24

**Authors:** Pathiyil Ravi Shankar

**Affiliations:** 1 IMU Center for Education, International Medical University, Kuala Lumpur, Malaysia

This is a well-written and presented article.

 Accreditation is important for maintaining quality assurance in education. Many accreditation agencies are in the process of getting themselves accredited by the World Federation for Medical Education (WFME). Hence this article is timely and important. Accreditation plays an important role in initiating, maintaining, and strengthening CQI processes at institutions.

 My suggestions for further improvement are:

**Major comments:**

 Table 1: Search Strategy Approach & Lore

 This section requires more description and explanation for readers to understand the strategies described.

 Discussion section: The authors have not described theme 1 Accreditation and its standards in general,

 The themes could be described in greater detail and the authors’ experiences can be incorporated.

 What are the limitations of this scoping review?

Are the rationale for, and objectives of, the Systematic Review clearly stated?

Yes

Is the statistical analysis and its interpretation appropriate?

I cannot comment. A qualified statistician is required.

Are sufficient details of the methods and analysis provided to allow replication by others?

Yes

Are the conclusions drawn adequately supported by the results presented in the review?

Yes

Reviewer Expertise:

Health professions education

I confirm that I have read this submission and believe that I have an appropriate level of expertise to confirm that it is of an acceptable scientific standard, however I have significant reservations, as outlined above.
